# Lipid droplet dynamics in metabolic regulation

**DOI:** 10.1039/d6cb00081a

**Published:** 2026-03-25

**Authors:** Ralf Weiskirchen, Sabine Weiskirchen, Amedeo Lonardo

**Affiliations:** a Institute of Molecular Pathobiochemistry, Experimental Gene Therapy and Clinical Chemistry (IFMPEGKC), RWTH University Hospital Aachen Pauwelsstr. 30 D-52074 Aachen Germany rweiskirchen@ukaachen.de sweiskirchen@ukaachen.de; b AOU Modena, Ospedale Civile di Baggiovara (-2023) Modena 41100 Italy a.lonardo@libero.it

## Abstract

Lipid droplets (LDs) are ubiquitous intracellular organelles that store neutral lipids such as triacylglycerols and sterol esters within a phospholipid monolayer decorated by a specialized proteome. Far from being inert depots, LDs are highly dynamic hubs that integrate lipid storage with cellular and systemic metabolic regulation. Their biogenesis, growth, remodeling, and catabolism are tightly controlled by nutrient status, hormonal signaling, and cellular stress, and are coupled to key metabolic pathways including β-oxidation, lipogenesis, membrane synthesis, and signaling lipid production. Aberrant LD dynamics coexist with a broad spectrum of metabolic pathologies, from obesity and insulin resistance to metabolic dysfunction-associated steatotic liver disease, lipodystrophies, and cancer. In this review, we discuss current concepts of LD biogenesis and expansion, cytosolic lipolysis and lipophagy, and the physical and functional interactions of LDs with mitochondria, peroxisomes, endoplasmic reticulum, and lysosomes. We highlight how tissue-specific LD biology in adipose tissue, liver, skeletal muscle, pancreatic β-cells, and immune cells shapes systemic energy homeostasis and the response to metabolic stress. Particular emphasis is placed on chemical biology and imaging approaches that have transformed our ability to visualize and manipulate LDs in space and time, including fluorescent lipid probes, metabolic labeling, organelle-targeted proximity labeling, lipidomics, and functional screening. Finally, we outline the opportunities and challenges in therapeutically targeting LD dynamics for metabolic disorders. This includes the emerging concept of exploiting LDs as drug and nucleic acid delivery platforms. We also discuss the outstanding questions that need to be addressed in order to safely use LD biology for clinical benefit.

## Abstract 2

Lipid droplets are dynamic organelles for lipid storage and metabolic signaling. This review summarizes their biogenesis and turnover, tissue-specific roles in metabolic disease, and therapeutic targeting opportunities.

## Graphical abstract

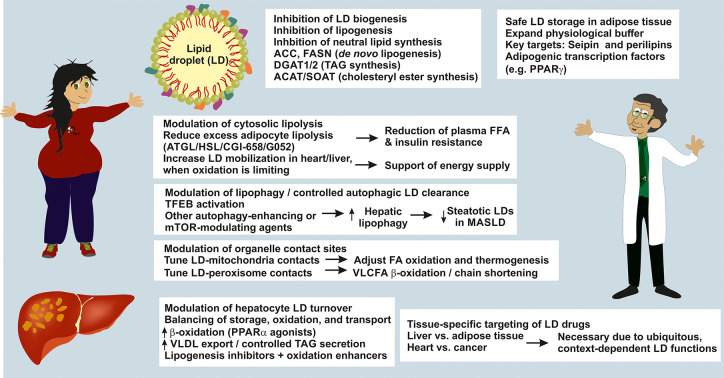

## Introduction

Lipid droplets (LDs) are evolutionarily conserved organelles that store neutral lipids across virtually all organisms, from bacteria and yeast to plants and mammals.^1–3^ Structurally, LDs consist of a hydrophobic core of triacylglycerols (TAGs) and sterol esters surrounded by a phospholipid monolayer and a selective protein coat.^1,4^ This unique architecture sets LDs apart from bilayer-bound organelles and underlies many of their distinctive biophysical and functional properties. Historically, LDs were seen as passive fat deposits that buffer fluctuations in fatty acid availability and provide fuel during fasting or increased energy demand. However, over the last two decades, this perception has fundamentally changed.^3^ LDs are now understood as dynamic organelles that continuously form, grow, shrink, move, and engage in regulated interactions with other compartments.^5^ Importantly, their surface serves as a platform for key enzymes in lipid metabolism as well as regulators of signaling, trafficking, and proteostasis.

By storing excess fatty acids as inert TAGs, LDs protect cells from lipotoxic species such as non-esterified fatty acids, diacylglycerols, and ceramides, which can disrupt membranes, trigger ER stress, and impair insulin signaling.^1^ Conversely, controlled mobilization of LD-stored lipids *via* cytosolic lipases and lipophagy is essential for fueling mitochondrial β-oxidation, sustaining membrane biosynthesis, generating lipid signaling mediators, and supporting anabolic demands during cell growth and proliferation.^1,3^ The term “LD dynamics” encompasses several interconnected processes: *de novo* biogenesis from the endoplasmic reticulum (ER), growth by local lipid synthesis and coalescence, protein and lipid remodeling of the LD surface, motility and organelle contacts, and degradation *via* lipolysis and autophagy.^1,6^ Each of these processes is tuned by nutrient and hormonal cues, including insulin, catecholamines, glucagon, and nuclear receptor signaling, and is subject to cell type-specific regulation.

Dysregulated LD dynamics are increasingly implicated in metabolic disease.^7,8^ In obesity and insulin resistance, excessive and poorly regulated LD storage in adipose tissue is accompanied by ectopic LD accumulation in the liver, skeletal muscle, and pancreatic β-cells. In metabolic dysfunction-associated steatotic liver disease (MASLD) and its progressive form, metabolic dysfunction-associated steatohepatitis (MASH), hepatic LDs can be both protective (by sequestering toxic lipids) and pathogenic (by promoting inflammation, fibrosis, and hepatocellular carcinoma when overload and lipotoxicity ensue).^1,9^ Conversely, in lipodystrophies, impaired LD biogenesis or expansion causes a failure of safe lipid storage, leading to severe systemic metabolic derangements despite a lack of obesity. Beyond classical metabolic disease, LD reprogramming contributes to the metabolic plasticity of cancer cells, supports viral replication, and shapes immune responses by controlling the synthesis of eicosanoids and other lipid mediators.^10^ These diverse roles highlight the need to understand LD biology at multiple scales, from the molecular mechanisms of protein and lipid sorting at the LD surface to organismal energy balance.

In the following sections, we will first outline the current models of LD biogenesis and growth. We will then describe the mechanisms of LD catabolism through lipolysis and lipophagy. Next, we will discuss how LD-organelle contact sites coordinate lipid flux and metabolic regulation. After that, we will examine tissue-specific LD functions that play a role in systemic metabolic homeostasis and disease. We will also highlight chemical biology and imaging tools that allow for quantitative analysis and manipulation of LD dynamics. Finally, we will conclude with perspectives on therapeutic targeting and address open questions in the field.

## Biogenesis and growth of lipid droplets

### *De novo* formation of lipid droplets at the endoplasmic reticulum

**Fig. 1:**
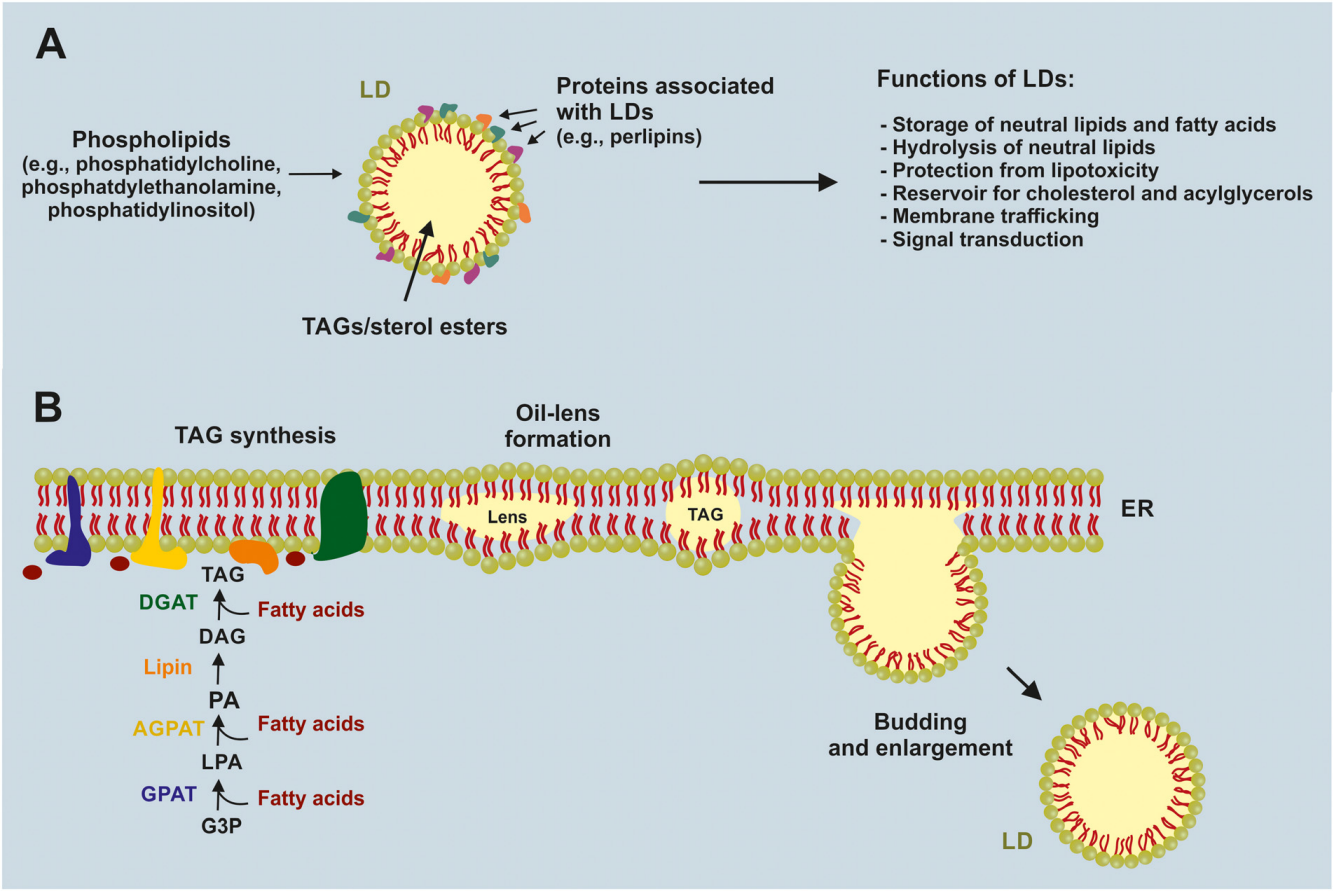
Structural organization and *de novo* biogenesis of lipid droplets (LDs). (A) Schematic representation of a cellular LD consisting of a core of neutral lipids, mainly triacylglycerols (TAGs) and sterol esters, surrounded by a phospholipid monolayer enriched in phosphatidylcholine (PC), phosphatidylethanolamine (PE) and phosphatidylinositol (PI), and coated with LD-associated proteins such as perilipins (PLINs). The text panel summarizes major LD functions, including storage and hydrolysis of neutral lipids and fatty acids, protection from lipotoxicity, serving as a reservoir for cholesterol and acyl-glycerols, and participation in membrane trafficking and signal transduction. (B) Model illustrating *de novo* LD biogenesis at the endoplasmic reticulum (ER). Glycerol-3-phosphate (G3P) is sequentially acylated by glycerol-3-phosphate acyltransferase (GPAT, blue) and 1-acylglycerol-3-phosphate acyltransferase (AGPAT, yellow) to produce lysophosphatidic acid (LPA) and phosphatidic acid (PA). Lipin (orange) then dephosphorylates PA to form diacylglycerol (DAG), which is finally converted to TAG by diacylglycerol acyltransferase (DGAT, green). Newly synthesized TAG accumulates between the ER membrane leaflets as neutral-lipid “oil lenses” that grow, bud toward the cytosol, and enlarge into mature LDs while initially remaining connected to the ER.

LD biogenesis is closely linked to neutral lipid synthesis in the ER.^11,12^ The final step of TAG synthesis is catalyzed by diacylglycerol acyltransferases (DGAT1 and DGAT2), which convert diacylglycerol and acyl-CoA to TAG (Fig. 1).^12^ Sterol *O*-acyltransferases (also known as acyl-CoA: cholesterol acyltransferases, ACAT/SOAT1 and SOAT2) esterify cholesterol to produce sterol esters.^12^ These neutral lipids accumulate within the hydrophobic core of the ER bilayer, forming oil-like lenses that grow between the two leaflets. Once they reach a critical size, these neutral lipid lenses bud from the cytosolic leaflet of the ER, forming nascent LDs. The process of budding is unique, as the LD remains connected to the cytosolic leaflet of the ER through a neck-like structure, maintaining the continuity of the phospholipid monolayer and allowing for ongoing lipid and protein exchange.^11–13^ The specific physical forces and protein machinery responsible for lens nucleation, growth, and budding are incompletely understood, but curvature-stabilizing lipids, local phospholipid composition, and specific protein scaffolds that include seipin are all implicated.^14,15^

### Protein factors controlling lipid droplet biogenesis

Several conserved protein families play critical roles in LD biogenesis (Table 1). The ER-resident protein seipin is essential for the formation of normal LDs in yeast, flies, and mammals. Loss-of-function mutations in the human seipin gene (*BSCL2*) cause congenital generalized lipodystrophy, demonstrating that seipin is required for appropriate LD formation in adipocytes and systemic metabolic health.^16^ Seipin is enriched at discrete ER sites that mark the emergence of nascent LDs and may function as a scaffold that organizes neutral lipid lenses and coordinates protein recruitment.^17^

**Table 1:** Selected proteins involved in lipid droplet biogenesis and early growth

Protein/family	Gene(s) (human)	Principal localization in LD pathway	Primary role in LD biogenesis/early growth	Selected features/physiological relevance	Key references
Core ER biogenesis factors					
Seipin	*BSCL2*	Discrete ER domains marking LD formation sites and ER-LD junctions	Scaffolds specialized ER sites where neutral lipid lenses nucleate and nascent LDs emerge, stabilizing the lens and coordinating protein recruitment to forming LDs.	Essential for the formation of normal LDs in yeast, flies, and mammals. Loss-of-function mutations in humans cause congenital generalized lipodystrophy, highlighting its central role in adipocyte LD formation and systemic metabolic health.	1, 11, 14 and 15
FIT (fat storage–inducing transmembrane) proteins	*FITM1, FITM2*	ER membrane	Bind and partition TAG within the ER bilayer, promoting the accumulation of neutral lipids on the cytosolic leaflet. This facilitates lens growth and budding towards the cytosol.	Modulate LD number and size by adjusting how efficiently TAG is retained and concentrated at LD budding zones on the ER.	11 and 12
DGAT1	*DGAT1*	Broadly distributed in ER membrane	Catalyzes the final step of TAG synthesis from diacylglycerol (DAG) and acyl-CoA in the ER, contributing to the neutral lipid pool that forms LD lenses.	Plays a less prominent role in TAG synthesis compared to DGAT2. Its contributions to LD biogenesis and expansion vary depending on the tissue and physiological state.	11, 12 and 18
DGAT2	*DGAT2*	ER-LD junctions and on the LD surface after budding	Enriched at ER-LD junctions, where it channels newly synthesized TAG directly into the nascent LD core, driving LD budding and early surface growth.	Spatial restriction to ER-LD contacts makes DGAT2 a key driver of localized TAG production and efficient LD formation in many settings.	1, 11 and 12
Sterol O-acyltransferases (ACAT/SOAT)	*SOAT1 (ACAT1), SOAT2 (ACAT2)*	ER membrane	Esterify free cholesterol to form sterol esters (SEs) that, along with TAG, accumulate in the ER bilayer and contribute to the formation of hydrophobic neutral lipid lenses.	Adjust the composition and volume of neutral lipids available for LD biogenesis. Changes in ACAT/SOAT activity can lead to alterations in the LD content of sterol esters compared to TAG.	11 and 12
ER-shaping proteins/reticulon family	*e.g.* RTN family	High-curvature ER tubules; LD budding hotspots	Generate and stabilize ER membrane curvature and topology at LD budding sites, influencing the location where neutral lipid lenses nucleate and how they bud towards the cytosol.	Proposed contributors to the physical forces and membrane geometry underlying lens nucleation, growth, and neck formation between ER and nascent LDs.	2, 5 and 11
Glycerol-3-phosphate acyltransferase (GPAT) isoforms	*e.g. GPAT3, GPAT4*	ER and LD surface	Initiate glycerolipid synthesis by acylating glycerol-3-phosphate. When recruited to LDs, they support local TAG synthesis at the LD surface for post-budding growth.	Coordination of GPAT activity with DGAT2 at LDs favors the enlargement of existing droplets and influences the distribution of LD number and size.	1 and 6
LD surface remodeling and growth factors					
CIDE proteins	*CIDEA, CIDEB, CIDEC/FSP27*	LD surface, enriched at LD-LD contact sites	Mediate the directional neutral lipid transfer between adjacent LDs at contact sites, promoting the coalescence of smaller LDs into fewer, larger droplets.	Major regulators of LD size distribution. In adipocytes, CIDE-dependent LD–LD fusion, which underlies the formation of large unilocular LDs characteristic of white fat.	1 and 5
Perilipin 1 (PLIN1)	*PLIN1*	LD surface in white and brown adipocytes	Stabilizes the LD surface and tightly regulates access of cytosolic lipases to the LD stored TAG, thereby influencing whether the LD expands or breaks down in response to hormonal cues.	In the fed state, non-phosphorylated PLIN1 restricts lipase access and promotes LD growth, while PKA-mediated phosphorylation during catecholamine stimulation allows ATGL/HSL access and impacts LD size and quantity.	1 and 7
Perilipin 2 (PLIN2)	*PLIN2*	LD surface in many non-adipose cells such as hepatocytes and macrophages)	Early LD-coating protein that promotes LD biogenesis and maintenance by stabilizing the phospholipid monolayer and aiding in the recruitment of metabolic enzymes.	Broadly expressed marker of LDs whose abundance correlates with LD content in the liver and other tissues, linking LD formation to steatosis and foam-cell phenotypes.	1 and 6
Perilipin 3 (PLIN3)	*PLIN3*	LD surface in various cell types	Participates in early LD coating and contributes to LD biogenesis and stabilization, often acting redundantly or cooperatively with PLIN2.	Supports LD formation in non-adipose tissues and is commonly used as a general LD marker in cell biology studies.	1 and 6
Perilipin 5 (PLIN5)	*PLIN5*	LD surface in oxidative tissues (heart, skeletal muscle, brown fat); LD-mitochondria contact sites	Coordinates LD maintenance and LD-mitochondria coupling by organizing complexes that link LD lipolysis to mitochondrial β-oxidation at contact sites.	High PLIN5 expression in oxidative tissues facilitates efficient utilization of LD-stored lipids for energy and influences LD size, turnover, and metabolic flexibility in muscle and heart.	1, 6 and 8
Other LD-surface enzymes (overview)	*e.g. DGAT2*, *GPAT* isoforms once on LD	LD surface after budding	Provide local neutral lipid synthesis directly at the LD monolayer, allowing for continued LD expansion without the need for bulk ER-to-LD transfer.	Redistribution of lipid-synthesizing enzymes from the ER to LDs is a crucial shift from *de novo* biogenesis to growth and remodeling of pre-existing droplets.	1, 11 and 12

Collectively, the proteins described above delineate two functionally coupled modules of LD formation: an ER-resident core biogenesis machinery and a set of LD-surface remodeling factors that act on nascent droplets after they emerge from the ER. Core ER factors such as seipin, FIT proteins, DGAT1/2, ACAT/SOAT, and ER-shaping proteins determine where neutral lipid lenses nucleate, how they grow within the ER bilayer, and how budding LDs remain topologically connected to the cytosolic leaflet.^12^ In contrast, LD-surface proteins, including CIDE family members and perilipins, primarily control post-budding growth, fusion, and stabilization of droplets, as well as the regulated access of metabolic enzymes to the LD monolayer.^19^ This division emphasizes that LD size, number, and composition are not solely dictated by the rate of neutral lipid synthesis in the ER, but also by subsequent remodeling events at the LD surface. Viewing LD biogenesis through this two-module framework provides a useful scaffold for interpreting how different genetic lesions or pharmacologic interventions can selectively impact lens nucleation, LD budding, or later stages of LD expansion and turnover in specific tissues.

Fat storage-inducing transmembrane (FIT) proteins are another family of ER factors that modulate LD formation, possibly by binding and partitioning TAG within the membrane and facilitating lens growth on the cytosolic side.^20^ Additional proteins, including some reticulon family members and ER-shaping proteins, may influence the local curvature and topology of LD budding sites. DGAT2 is often enriched at ER-LD junctions, where it can directly channel newly synthesized TAG into the LD core, whereas DGAT1 tends to reside more broadly within the ER. The relative roles of DGAT1 and DGAT2 in LD biogenesis and expansion vary between tissues and physiological states, reflecting differences in expression and substrate specificity.^21^

### Lipid droplet expansion and remodeling

Once formed, LDs can significantly increase in size.^6^ Two non-exclusive mechanisms contribute to LD growth. In the first, LDs enlarge through local synthesis of TAG at their surface, supported by the recruitment of enzymes such as DGAT2 and glycerol-3-phosphate acyltransferase isoforms. In the second, LDs grow through coalescence and fusion of smaller droplets, a process regulated by members of the cell death-inducing DFF45-like effector (CIDE) protein family.^6^ CIDE proteins localize to LD-LD contact sites and facilitate the directional transfer of neutral lipids from one LD to another, resulting in the formation of fewer but larger droplets.^22^

The surface of LDs is coated by a family of proteins known as perilipins (PLIN1-PLIN5 in mammals), which play a central role in LD stability and metabolic regulation.^23^ Different perilipins are expressed in a tissue-specific manner and confer distinct properties. For example, PLIN1 is predominantly found in adipocytes and regulates the access of lipases to LD TAGs, while PLIN2 and PLIN3 are more widely expressed and contribute to LD biogenesis and maintenance in various cell types. PLIN4 is mainly expressed in adipose tissue and certain skeletal muscle fibres, where it decorates nascent and expanding LDs and cooperates with other perilipins in controlling LD size and stability. In adipocytes, PLIN4 is particularly enriched on larger droplets during differentiation, linking it to LD growth and the transition towards unilocular fat storage. PLIN5 is abundant in oxidative tissues like the heart and skeletal muscle, promoting functional interaction between LDs and mitochondria.^23^ The phospholipid composition of the LD surface also influences LD dynamics. Phosphatidylcholine (PC) is the primary monolayer component and acts as a surfactant that stabilizes LDs.^24^ Inadequate PC relative to neutral lipids can lead to LD coalescence and the formation of larger droplets, while alterations in other lipids such as phosphatidylethanolamine, phosphatidic acid, and lysophospholipids can affect LD curvature, protein recruitment, and the likelihood of fusion or fission.^24^

### Nutrient and hormonal regulation of lipid droplet formation

LD biogenesis and expansion are closely linked to nutrient availability and hormonal signaling. In conditions of caloric excess and high circulating lipid levels, lipogenic transcription factors such as sterol regulatory element-binding proteins (SREBPs) and carbohydrate-responsive element-binding protein (ChREBP), along with nuclear receptors like peroxisome proliferator-activated receptors (PPARs) and liver X receptors (LXRs), drive the expression of genes involved in fatty acid uptake, *de novo* lipogenesis, and TAG synthesis. This coordinated response promotes LD formation and expansion to safely store excess energy.^25^ Insulin signaling further stimulates lipogenesis in adipose tissue and liver by activating Akt and mTOR pathways, enhancing glucose uptake and anabolic metabolism.^3,26^ In adipocytes, insulin also suppresses lipolysis, promoting net TAG accumulation in LDs. Conversely, during fasting or catecholamine stimulation, lipogenesis is inhibited and lipolysis is activated, leading to LD shrinkage and increased lipid flux out of LDs to support energy production. Recently, interferon regulatory factor-2 binding protein 2 (IRF2BP2) was identified as a transcriptional repressor of adipocyte lipolysis.^27^ In non-adipose tissues, LD formation can be induced by exposure to excess fatty acids, particularly saturated species, as a way to sequester potentially toxic lipids. In these cases, LD biogenesis often serves as a protective response that buffers acute lipid overload, although chronic exposure may result in pathological lipid accumulation and organ dysfunction.^28^

## Catabolism of lipid droplets: lipolysis and lipophagy

### Cytosolic lipolysis and hormone-sensitive regulation

The classical pathway for LD catabolism is cytosolic lipolysis, which involves the stepwise hydrolysis of TAG to glycerol and free fatty acids (FFAs) by a series of lipases.^29^ The rate-limiting enzyme for TAG breakdown is adipose triglyceride lipase (ATGL), which hydrolyses TAG to diacylglycerol (DAG) and FFAs. Hormone-sensitive lipase (HSL) primarily converts DAG to monoacylglycerol (MAG), while monoacylglycerol lipase (MGL) hydrolyses MAG to glycerol and FFAs.^29^

In adipocytes, these enzymes are under tight hormonal control. During fasting or stress, catecholamines activate β-adrenergic receptors, leading to cAMP production and protein kinase A (PKA) activation.^30^ PKA phosphorylates both perilipin 1 and HSL. Phosphorylated Perilipin 1 undergoes conformational changes that permit access of ATGL to the LD surface and promote HSL translocation from the cytosol to LDs. PKA-mediated phosphorylation of HSL also increases its catalytic activity.^30^ Together, these events trigger robust lipolysis, releasing FFAs into the circulation for uptake and oxidation by other tissues. Insulin exerts the opposite effect by activating phosphodiesterases that degrade cAMP and by promoting phosphatase-mediated dephosphorylation of perilipin and HSL, thereby suppressing lipolysis. Insulin also enhances FFA re-esterification and LD TAG synthesis, maintaining net lipid storage under fed conditions.^31^

In non-adipose tissues, the same core lipolytic machinery is present but is regulated by distinct perilipin isoforms and tissue-specific co-factors. PLIN2 and PLIN3, for example, are abundant on LDs in hepatocytes, macrophages, and many other cell types, while PLIN5 plays a key role in oxidative tissues by coordinating LD lipolysis with mitochondrial β-oxidation.^32^ ATGL co-activators and inhibitors, such as comparative gene identification-58 (CGI-58) and G0/G1 switch gene 2 (G0S2), further fine-tune lipolytic activity in a cell type- and context-dependent manner.^33,34^

### Lipophagy and autophagic degradation of lipid droplets

In addition to cytosolic lipases, LDs are degraded through autophagy, a process known as lipophagy. In macroautophagy, double-membrane autophagosomes engulf LDs or LD fragments and merge with lysosomes. Within lysosomes, lysosomal acid lipase breaks down neutral lipids.^35^ Microautophagy and selective forms of autophagy can also play a role in LD turnover, such as the direct invagination of lysosomal membranes around LD portions. Lipophagy is especially crucial during nutrient deprivation, as the autophagic breakdown of LDs provides FFAs for mitochondrial oxidation and supports ATP production.^36^ In hepatocytes, inhibiting autophagy leads to LD accumulation and steatosis, while enhancing lipophagy can decrease hepatic lipid content. In other cell types like macrophages and cardiomyocytes, lipophagy helps maintain lipid quality and energy balance.^35^ The selective recognition of LDs by the autophagic machinery involves LD-coating proteins and autophagy receptors that connect LDs to LC3-positive membranes.^36^ Suppressing LC3 leads to decreased LD formation in mammalian cells.^37^ Perilipins can either hinder or promote lipophagy depending on their phosphorylation and ubiquitination status.^32^ Ubiquitin ligases and adaptor proteins may target specific LD proteins for autophagic sequestration, thereby influencing the efficiency and selectivity of lipophagy.^38^

### Integration of lipolysis and lipophagy

Cytosolic lipolysis and lipophagy are not independent pathways, but rather interconnected and often coordinated.^39^ For example, partial lipolysis can produce smaller LDs that are more easily engulfed by autophagy, while lipophagy can enhance or compensate for decreased cytosolic lipase activity. Transcription factors like PPARs, TFEB, and members of the FOXO family co-regulate genes involved in both lipolysis and autophagy in response to nutrient and hormonal signals.^40,41^ The extent to which lipolysis and lipophagy contribute to LD breakdown varies depending on the tissue and physiological state. In adipocytes, cytosolic lipolysis is more prominent during acute hormone-induced lipolysis, whereas lipophagy may have a larger role in long-term LD storage remodeling. In the liver and other non-adipose tissues, lipophagy is a key factor in LD turnover during fasting, stress, and disease. Understanding how cells dynamically distribute LD degradation between these pathways is crucial for unraveling LD-mediated metabolic regulation.^42^

### Organelle contacts and metabolic integration

**Fig. 2:**
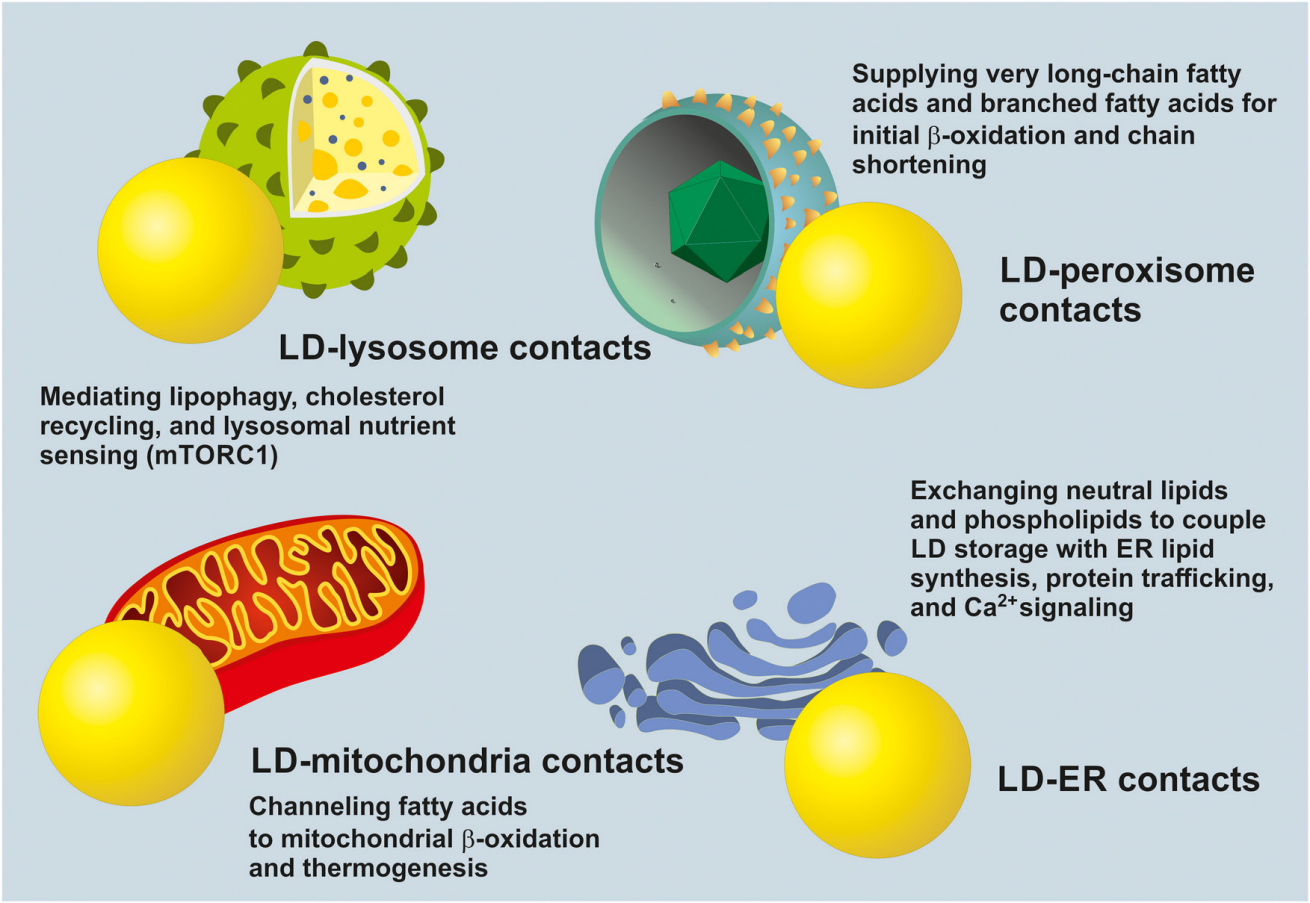
Lipid droplet-organelle contact sites coordinate lipid flux and metabolic regulation. Lipid droplets (LDs) form physical contact sites with mitochondria, peroxisomes, the endoplasmic reticulum (ER), and lysosomes that channel fatty acids and other lipids between storage and distinct metabolic fates. LD-mitochondria contact sites, enriched in tethering and signaling proteins such as PLIN5, promote efficient transfer of fatty acids released from LDs to mitochondrial β-oxidation and, in brown and beige adipocytes, support thermogenesis. LD-peroxisome contacts enable trafficking of very-long-chain and branched-chain fatty acids from LDs to peroxisomes for initial β-oxidation and chain shortening, after which partially oxidized acyl chains can be further degraded in mitochondria. Persistent LD-ER contacts maintain the continuity of neutral lipid and phospholipid exchange, mediate the targeting of LD-associated proteins, and integrate LD storage with ER-based membrane lipid synthesis, protein folding, and calcium signaling. LD-lysosome interactions support lipophagy and can modulate lysosomal nutrient-sensing pathways such as mTORC1. Cholesteryl esters hydrolyzed in lysosomes can be re-esterified and stored in LDs, illustrating the bidirectional nature of lipid traffic between these organelles.

LDs are embedded in an extensive interaction network with mitochondria, peroxisomes, ER, and lysosomes. These physical contact sites have emerged as key hubs that channel fatty acids and other lipids between storage and diverse metabolic fates (Fig. 2). By positioning LDs next to oxidative organelles, contact sites facilitate efficient β-oxidation while minimizing cytosolic accumulation of lipotoxic intermediates. In specialized contexts such as brown adipose tissue, LDs support thermogenesis. Persistent LD-ER contacts integrate neutral lipid storage with phospholipid synthesis, protein trafficking, and calcium signaling. LD-lysosome interfaces enable lipophagy and impinge on lysosomal nutrient-sensing pathways such as mechanistic target of rapamycin complex 1 (mTORC1). Together, these highly regulated and often tissue-specific LD-organelle junctions provide a spatial framework for coordinating lipid flux with cellular energy demands and stress responses.

### Lipid droplet-mitochondria interactions and β-oxidation

LDs establish close physical contacts with mitochondria, facilitating the channeling of FFAs for β-oxidation.^43,44^ In oxidative tissues such as the heart, skeletal muscle, and brown adipose tissue, LD-associated mitochondria are densely packed around LDs, and LD-mitochondria contact sites are enriched in specific tethering proteins and lipid transfer machinery.^45,46^ These contacts enable the efficient coupling of LD lipolysis to mitochondrial FFA uptake and oxidation, minimizing the accumulation of cytosolic FFAs and limiting lipotoxicity.^47,48^ LD-mitochondria coupling also supports thermogenesis in brown adipose tissue, where β-oxidation in mitochondria fuels uncoupling protein-mediated heat production. Conversely, the disruption of LD-mitochondria contacts can impair oxidative metabolism and contribute to the accumulation of lipotoxic intermediates that interfere with insulin signaling. PLIN5 is a key mediator of LD-mitochondria interactions in many tissues.^49^ It localizes at LD-mitochondria contact sites and organizes protein complexes that coordinate ATGL activity with mitochondrial metabolism. Additional tethers, including some mitofusins and other organelle contact site proteins, are also implicated in fine-tuning cellular bioenergetics, although their precise roles and regulation remain under active investigation.^50^

### Lipid droplet-peroxisome and lipid droplet-lysosome contacts

Peroxisomes complement mitochondria in fatty acid oxidation, especially for very-long-chain and branched-chain fatty acids.^44,51^ LD-peroxisome contact sites facilitate the transfer of these substrates from LDs to peroxisomes for initial chain shortening. Afterward, partially oxidized products can be further degraded in mitochondria. Proteins that simultaneously associate with LDs and peroxisomes contribute to the formation and regulation of these contacts. LDs also interact with lysosomes beyond the context of classical lipophagy. Stable LD-lysosome contacts can regulate lipid exchange and signaling.^52^ For example, lysosomal nutrient-sensing pathways, and lipid sorting functions, such as those involving mTORC1, respond to the availability of lipids and other nutrients. LD-lysosome interactions may influence these signaling axes.^53^ Low-density lipoprotein (LDL)-derived cholesteryl esters can be hydrolyzed in lysosomes, with free cholesterol and fatty acids subsequently redirected to LDs for storage, highlighting bidirectional traffic between these organelles.

### Lipid droplet-endoplasmic reticulum contacts and membrane lipid homeostasis

LDs remain functionally connected to the ER even after budding, both through their site of origin and additional contact sites.^47,54^ These LD-ER contacts are crucial for phospholipid exchange, protein trafficking, and integrating LD metabolism with ER functions such as lipid synthesis, protein folding, and calcium signaling. Recent studies using high resolution imaging have shown that a multimeric complex containing extended synaptotagmin (ESYT)1, ESYT2, and VAMP associated protein B (VAPB) and C are localized at the interface of LD-mitochondria-ER and are required to transfer fatty acids to enable β-oxidation.^55^

Through LD-ER connections, cells can dynamically adjust the balance between neutral lipid storage and membrane lipid production.^47,54^ For example, under conditions of increased membrane demand, TAG stored in LDs can be mobilized and re-esterified into phospholipids in the ER. Conversely, when excess fatty acids are present, *de novo* lipogenesis and neutral lipid synthesis in the ER supply LDs to prevent membrane perturbation and ER stress. LD-ER contact sites are enriched in lipid transfer proteins, including members of the oxysterol-binding protein-related and vacuolar protein sorting 13 (VPS13) families, which can shuttle lipids between LDs and the ER or other organelles.^55^ Regulation of these transfer pathways influences the composition and size of LDs and contributes to cellular adaptation to metabolic challenges.

## Tissue-specific roles of lipid droplet dynamics in systemic metabolic regulation

### Adipose tissue: central hub of energy storage and release

White adipose tissue is the primary site of long-term energy storage in mammals.^56^ Mature white adipocytes are characterized by a large unilocular LD that occupies most of the cytoplasmic volume, surrounded by a thin rim of cytoplasm containing the nucleus and organelles. Efficient LD storage in adipocytes allows other tissues to be protected from lipid overload. Adipocyte LD dynamics are specialized for rapid, hormone-controlled transitions between storage and mobilization. In the fed state, insulin promotes triglyceride (TAG) synthesis and LD expansion by stimulating glucose uptake, lipogenesis, and esterification of circulating FFAs. In fasting or stress, catecholamine-induced lipolysis mobilizes LD TAGs, releasing FFAs and glycerol into the circulation to fuel other tissues. The delicate balance between these states is crucial for maintaining systemic energy homeostasis.

Adipose tissue also acts as an endocrine organ, secreting adipokines such as leptin, adiponectin, and resistin, which modulate appetite, insulin sensitivity, and inflammation.^56,57^ LD size, number, and lipid composition in adipocytes influence adipokine production and adipose tissue inflammation, linking LD dynamics to whole-body metabolic regulation. Brown and beige adipocytes possess multilocular LDs and a high mitochondrial content specialized for thermogenesis.^56,57^ In these cells, tight coupling between LD lipolysis and mitochondrial β-oxidation underlies non-shivering heat production. The recruitment of beige adipocytes in white depots (browning) increases LD-mitochondria interactions and enhances energy expenditure, offering potential therapeutic avenues for obesity.

### Liver: balancing lipid storage, oxidation, and export

The liver plays a central role in integrating carbohydrate, lipid, and amino acid metabolism and has an enormous capacity for regeneration.^58^ Hepatocytes feature numerous small LDs whose abundance and size are highly responsive to nutritional, metabolic state, and hormonal cues.^58^ In the postprandial state, excess carbohydrates are converted to fatty acids *via de novo* lipogenesis, and hepatocyte LDs transiently expand. LD-stored TAGs can later be packaged into very-low-density lipoproteins (VLDL) for export, oxidized in mitochondria and peroxisomes, or retained as intrahepatic stores.^59^ Disruption of this balance leads to hepatic steatosis. Excessive LD accumulation in hepatocytes is a hallmark of MASLD, which can progress to MASH, cirrhosis, and hepatocellular carcinoma in some individuals.^60^ While LD formation initially serves as a protective mechanism to sequester toxic lipids, chronic overload, impaired LD turnover, and accumulation of lipotoxic lipid species can trigger inflammation, cell death, and fibrosis. Hepatic LD dynamics are influenced by systemic factors such as insulin, glucagon, and adipokines, as well as by intrinsic regulators including hormones, nuclear receptors, and transcriptional co-activators.^60^ Crosstalk between hepatocyte LDs and lipoprotein metabolism, bile acid synthesis, and glucose homeostasis further couples hepatic LD biology to whole-body metabolic regulation.

### Skeletal muscle: lipid droplets as fuel depots and the athlete's paradox

Skeletal muscle contains intramyocellular LDs (IMLDs) that serve as a local energy source for contraction, especially during endurance exercise. Endurance-trained athletes have a high content of IMLDs, yet they maintain excellent insulin sensitivity, a phenomenon known as the “athlete's paradox”.^61,62^ In contrast, individuals with obesity or type 2 diabetes often show elevated IMLDs linked to insulin resistance.^8,63^ Resolving this paradox involves considering not only the quantity of LDs but also their subcellular location, protein coating, and metabolic environment. In trained muscle, LDs are mainly small, abundant, and closely linked to mitochondria, with a high expression of oxidative LD proteins such as PLIN5.^8,61,64^ The coupling of LDs with mitochondria enables efficient oxidation of FFAs during exercise, preventing the accumulation of harmful compounds. In insulin-resistant muscle, LDs are typically larger, less densely distributed, and less tightly linked to mitochondria. This impaired interaction between LDs and mitochondria, along with reduced oxidative capacity and altered LD protein composition, promotes the accumulation of lipid intermediates that can disrupt insulin signaling.^63^ Therefore, the dynamics of LDs, rather than their static mass, are crucial factors in determining muscle metabolic health.

### Pancreatic β-cells and other tissues

Pancreatic β-cells are typically low in LD content under normal conditions but can accumulate LDs when exposed to chronic lipid excess and in patients suffering from type 2 diabetes.^65,66^ In the short term, LD formation may protect β-cells from lipotoxicity by sequestering FFAs as TAG. However, prolonged lipid exposure and dysregulated LD turnover can impair β-cell function, contributing to defects in glucose-stimulated insulin secretion and β-cell survival. LD dynamics also influence metabolism in other tissues, including the heart, kidney, and brain.^8^ Balanced LD formation and lipolysis are necessary to match fatty acid supply with oxidative demand, as both LD deficiency and LD overload can be harmful. In the heart, PLIN5 deficiency disrupts the energy metabolism homeostasis of cardiomyocytes, exacerbating pressure overload-induced cardiac hypertrophy and failure through oxidative stress.^8^ In renal cells and podocytes, LD accumulation is associated with diabetic nephropathy and other forms of kidney injury.^67^ In the central nervous system, emerging evidence suggests that neuron–glia interactions involving LDs play roles in lipid and elevated reactive oxygen species detoxification and neuroprotection.^68,69^ Immune cells, such as macrophages and neutrophils, rely on LDs as sources of arachidonic acid and other precursors for eicosanoid synthesis, contributing to foam cell formation in atherosclerotic plaques.^70,71^ LD accumulation in macrophages contributes to foam cell formation in atherosclerotic plaques, while LD dynamics in immune cells can modulate inflammatory responses and host defense against pathogens.

## Lipid droplets in metabolic disease and pathophysiology

### Obesity and insulin resistance

Obesity is defined as an increased mass of adipose tissue, caused by both the enlargement of fat cells (hypertrophy) and an increase in the number of fat cells (hyperplasia). A consistently positive energy balance contributes to the continuous growth of fat cells.^72^ Over time, adipose tissue can become dysfunctional, leading to hypoxia, fibrosis, and inflammation in expanding fat stores. When the storage capacity of adipose tissue is exceeded or impaired, fats can accumulate in other tissues, such as the liver, skeletal muscle, heart, bones, and pancreatic β-cells. Initially, this accumulation of fat droplets may be a protective mechanism, but long-term overload and impaired LD turnover can result in the buildup of harmful lipids that disrupt insulin signaling and organ function.^73^ Changes in the expression or function of LD proteins and enzymes, such as perilipins, ATGL, and CGI-58, have been associated with insulin resistance in both humans and animal models.^74^

### Metabolic dysfunction-associated steatotic liver disease and metabolic dysfunction-associated steatohepatitis

MASLD encompasses a spectrum from simple steatosis, characterized by an excessive accumulation of hepatic LDs, to MASH, which also involves inflammation, hepatocyte injury, and often variable stages of fibrosis. MASH, compared to uncomplicated steatosis, poses a higher risk of progressing to cirrhosis and hepatocellular carcinoma in a proportion of cases.^75^ LD-focused mechanisms play a role in various stages of MASLD development. Increased hepatic lipid uptake, heightened *de novo* lipogenesis, decreased VLDL secretion, and impaired LD catabolism all contribute to hepatic LD enlargement. While storing lipids in LDs helps reduce levels of harmful FFAs and other toxic intermediates, prolonged imbalance can lead to ongoing ER stress, oxidative stress, and mitochondrial dysfunction.^76^ The dysregulation of lipophagy is particularly significant. Studies in animals have shown that inadequate lipophagy can exacerbate steatosis, while excessive or uncontrolled autophagy may lead to cell death and inflammation.^77^

**Fig. 3:**
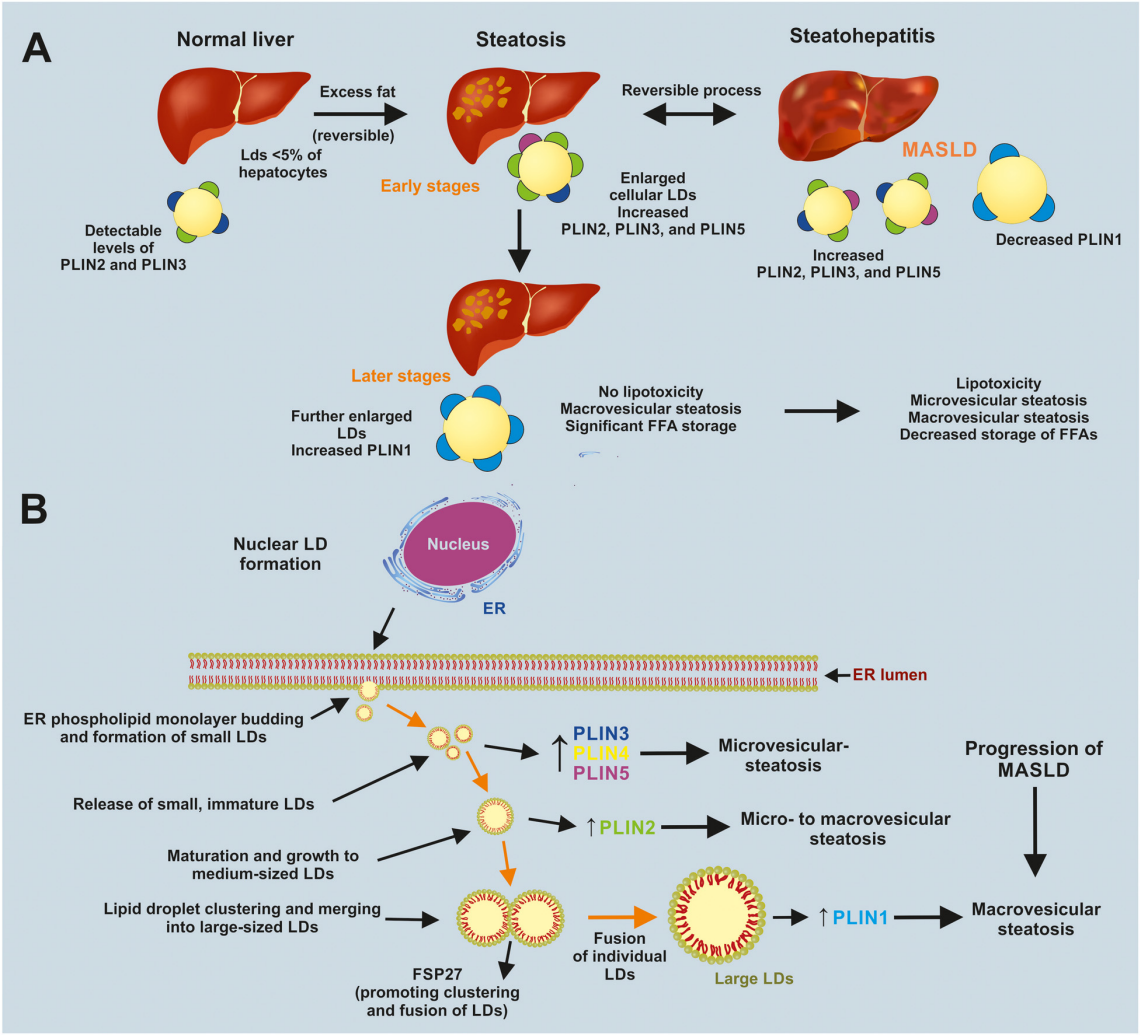
Perilipin remodeling of hepatic lipid droplets during progression from normal liver to metabolic dysfunction-associated steatotic liver disease (MASLD). (A) In a normal liver, only a minority of hepatocytes contain small lipid droplets (LDs) with low levels of PLIN2 and PLIN3. Chronic excess fat exposure induces steatosis with enlarged LDs that show increased coating by PLIN2, PLIN3, and PLIN5, supporting neutral lipid storage, limiting lipotoxicity, and remaining largely reversible. At later stages of steatosis, LDs further enlarge and PLIN1 expression rises, favoring extensive triacylglycerol storage and macrovesicular steatosis without overt lipotoxicity. Progression to steatohepatitis within the MASLD spectrum is associated with heterogeneous LD populations, persistent elevation of PLIN2/3/5 but relative loss or dysregulation of PLIN1 on subsets of droplets, a mixture of micro- and macrovesicular steatosis, reduced safe storage capacity for free fatty acids (FFAs), and the emergence of lipotoxic injury. (B) Schematic model of LD biogenesis and growth at the nuclear envelope and endoplasmic reticulum (ER) during steatosis. Neutral lipid synthesis drives budding of a phospholipid monolayer to form small nascent LDs enriched in PLIN3, PLIN4, and PLIN5, which are released as immature droplets that mature into medium-sized LDs with increased PLIN2. These LDs subsequently cluster and fuse through the action of the CIDE family protein FSP27 (CIDEC), generating large unilocular LDs characteristic of macrovesicular steatosis that acquire high PLIN1 levels. Dynamic changes in PLIN isoform composition (elevated PLIN2/3/5 on microvesicular droplets and PLIN1 on large droplets) accompany the transition from micro- to mixed-type and macrovesicular steatosis along the MASLD disease continuum (historically referred to as non-alcoholic fatty liver disease (NAFLD) in some classifications).

Perilipins play a crucial role in regulating the behavior of hepatic LDs in altered lipid homeostasis leading to MASLD and MASH.^76^ They help determine whether neutral lipid storage remains protective or becomes a source of lipotoxic stress. PLIN2 and PLIN3 are LD-coating proteins that are widely expressed and their levels closely mirror content in hepatocytes. In steatotic livers, their abundance is consistently increased, supporting LD biogenesis and stabilization, thereby serving as a biomarker of disease severity (Fig. 3). PLIN5, typically found in oxidative tissues, is also induced in fatty liver. It promotes the interaction between LDs and mitochondria, facilitating fatty acid channeling to β-oxidation and preventing the accumulation of toxic lipid intermediates in early MASLD.76 On the other hand, PLIN1 is usually low in hepatocytes but can be expressed on macrovesicular LDs during advanced steatosis. It restricts cytosolic lipase access, favoring continued TAG storage over lipolysis. In early disease stages, a PLIN2/3/5-dominated LD coat may help sequester and oxidize excess fatty acids. As the disease progresses, a shift towards PLIN1-rich, hypertrophic LDs may preserve neutral lipid storage but limit metabolic flexibility. As steatohepatitis develops, there are likely changes in PLIN expression. PLIN2/3/5 may persist on smaller, more lipase-accessible droplets, while PLIN1 may be lost or dysregulated on others. This reduces the capacity for safe fatty acid storage, increases lipolysis, and promotes inflammation, cell death, and fibrogenesis. These findings underscore the importance of perilipins in LD function in MASLD/MASH and suggest that they could be potential target for therapeutic intervention in hepatic lipid storage and turnover.^76^

Understanding how to regulate LD dynamics in hepatocytes to maintain protective storage while minimizing lipotoxicity is a critical challenge for developing therapies targeting MASLD and MASH.

### Lipodystrophies and lipid droplet biogenesis defects

Lipodystrophies are rare heterogeneous disorders that can be genetic or acquired. They are characterized by selective or generalized loss of adipose tissue and can lead to severe metabolic complications such as insulin resistance, hypertriglyceridemia, hepatic steatosis, and diabetes.^78^ Many congenital lipodystrophies are caused by mutations in genes that encode proteins essential for the biogenesis and maintenance of LDs in adipocates, such as seipin (encoded by *BSCL2*) and select perilipins or lipases.^79^ In these conditions, the defective formation of LDs in adipocytes significantly reduces the capacity for safe lipid storage. Consequently, lipids are redirected to non-adipose tissues, where ectopic LD accumulation and lipotoxicity can cause mitochondrial dysfunction, affecting organ homeostasis.^80^ These human genetic syndromes highlight the critical importance of proper LD biogenesis and dynamics for overall metabolic health.

### Atherosclerosis, cancer, and other conditions

In atherosclerosis, macrophages in the arterial wall take up modified lipoproteins and accumulate cholesteryl ester-rich LDs, becoming foam cells. The pathology can be roughly divided into three stages: (i) lipid-streak stage, (ii) fibrous plaque stage, and (iii) advanced lesions and thrombosis.^81^ The balance between LDL uptake, cholesteryl ester formation and hydrolysis, efflux of free cholesterol, and LD turnover determines plaque lipid burden and stability. Modulating LD dynamics in macrophages and vascular cells may thus influence atherogenesis and plaque regression.

Cancer cells frequently remodel LD metabolism to support rapid proliferation and survival in nutrient-poor or hypoxic microenvironments.^82^ LDs provide a reservoir of fatty acids for membrane synthesis, energy production, and signaling; they also buffer lipotoxic stress and help maintain redox balance. Elevated *de novo* lipogenesis, increased exogenous fatty acid uptake, and altered LD lipolysis are common features of tumor cell metabolism.^82^ In some cancers, high LD content correlates with increased tumor aggressiveness and therapy resistance.^83,84^ Similar to cancer, LD reprogramming is also observed in viral infection, neurodegeneration, and other diseases in humans and animals, underscoring the diverse pathophysiological contexts in which LD dynamics intersect with metabolic regulation.^85–87^

### Chemical biology and methods to study lipid droplet dynamics

Recent advances in chemical biology, imaging, and omics have provided a powerful toolkit for investigating LD dynamics with high spatial and temporal resolution, as well as for connecting LD behavior to cellular and systemic metabolic regulation. Table 2 summarizes various chemical biology and imaging approaches commonly used to visualize LDs, determine their molecular composition, and analyze their interactions with other organelles over time and space. The table covers neutral lipid dyes, metabolically integrated lipid probes, genetically encoded LD markers, advanced microscopy techniques, and proximity-labeling methods like APEX, BioID, and TurboID, which allow for mapping of LD-associated proteomes and contact site interactomes *in situ*. Additionally, it demonstrates how LD isolation, lipidomics, and high-content imaging can be integrated with genetic and pharmacological interventions to measure LD phenotypes and lipid fluxes under specific metabolic conditions. Together, these complementary methods offer a flexible toolkit for investigating LD dynamics at both the molecular and systems levels.

**Table 2:** Overview of experimental approaches to label lipid droplets and map their interactions

Method	Principle	Readouts	Typical applications	Advantages	Limitations
Neutral lipid-selective fluorescent dyes (Nile Red, Oil Red O, BODIPY 493/503, and newer BODIPY LD probes)	Hydrophobic dyes preferentially partition into the neutral lipid cores (TAG, sterol esters) of LDs in fixed or live cells.	LD number, size, morphology and subcellular distribution; global neutral lipid burden under different metabolic or pharmacological conditions.	Rapid screening of LD accumulation in response to nutrient overload, lipotoxic stress, or genetic/chemical perturbations; baseline LD phenotyping in diverse cell types and tissues.	Simple, inexpensive, and compatible with standard widefield/confocal microscopes; suitable for live-cell imaging and high-content screening; some probes are optimized for multicolor and super-resolution imaging.	Limited chemical specificity (TAG vs sterol esters); can label other neutral lipid pools; signal is only semi-quantitative; potential photobleaching and phototoxicity in long time-lapse experiments.
Metabolic fluorescent lipid probes (BODIPY-fatty acids, alkyne FAs with click chemistry, fluorescent TAG mimetics, and photoactivatable lipids)	Exogenous labeled fatty acids or lipid analogues are taken up and incorporated into endogenous lipid metabolism, including esterification into TAG and storage in LDs. Alkyne-tagged lipids are detected post-fixation *via* click chemistry.	Kinetics of lipid uptake, esterification and incorporation into nascent *vs.* pre-existing LDs; LD formation, growth and turnover in pulse-chase experiments.	Quantifying LD biogenesis and breakdown; tracking how specific fatty acids are stored in LDs *versus* rerouted to membranes or β-oxidation; dissecting LD dynamics under fasting/feeding, stress, or drug treatment.	Enables temporal resolution (pulse-chase) and tracking of defined lipid species; compatible with multiplexed imaging; photoactivatable probes provide spatiotemporal control of lipid release and LD labeling.	Labeled fatty acids and lipid mimetics may not fully mimic native lipids, can show biased partitioning into specific metabolic fates, and at high concentrations can perturb normal lipid metabolism and trafficking. Click-chemistry-based probes generally require fixation and may incompletely report on total lipid flux, complicating quantitative interpretation.
Fluorescent protein-tagged LD proteins (*e.g.* PLIN1-5, DGAT2, and GPAT4 fusions)	LD-resident proteins (perilipins, DGAT2, GPAT isoforms) are expressed as fusions with GFP/mCherry or other FPs, decorating the LD phospholipid monolayer in live cells.	LD surface morphology, protein composition and dynamics; recruitment/dispersion of specific LD proteins during lipolysis, lipophagy, or organelle contact formation.	Live-cell tracking of LD motility and fusion/fission; monitoring how PLINs, lipases, and biosynthetic enzymes remodel the LD surface; co-localization with mitochondria, ER, peroxisomes and lysosomes	With high specificity for LD surface, it allows advanced live-cell assays (FRAP, single-particle tracking) and can distinguish subpopulations of LDs by protein coat.	Overexpression can alter LD size, number, or lipolysis; requires genetic manipulation and validation that fusion proteins behave like endogenous counterparts.
Confocal/spinning-disk imaging of LD-organelle contacts	LDs are labeled (dyes or FP–LD markers) and combined with fluorescent markers for mitochondria, ER, peroxisomes or lysosomes to visualize close apposition and dynamic contacts in live cells.	Spatial proximity and apparent contact frequency between LDs and other organelles; gross dynamics of contact formation and dissolution over time.	Studying LD-mitochondria coupling in oxidative tissues, LD-ER continuity during biogenesis, and LD-lysosome interactions during lipophagy.	Direct visualization of LD-organelle relationships in intact cells; compatible with time-lapse imaging and pharmacological or genetic perturbations.	Resolution is diffraction-limited, so “contact” is inferred from overlap/adjacency; cannot resolve nanoscale organization within contact sites; requires careful colocalization quantification.
Super-resolution microscopy (STED, PALM, STORM) with LD dyes or FP markers	Application of super-resolution optical methods to LD labels and organelle markers improves spatial resolution far below the diffraction limit.	Nanoscale organization of proteins on LD surfaces and at LD-organelle contact zones (*e.g.* clustering of PLINs, tethering factors, lipases).	Mapping how LD-surface proteins and tethers are arranged at LD-mitochondria, LD-ER, or LD-lysosome interfaces; resolving structural changes during LD biogenesis or lipophagy.	Reveals fine structure of LD surfaces and contact sites not accessible by conventional light microscopy.	Technically demanding; limited field of view and throughput; live-cell super-resolution is challenging and can be phototoxic.
CLEM (correlative light and electron microscopy)	LDs and organelles are first imaged by fluorescence, then the same cells/regions are examined by EM (TEM or FIB-SEM) to link fluorescent signals to ultrastructure.	Precise ultrastructural context of fluorescently labeled LDs, including ER neck connections, LD-mitochondria contacts, and lysosomal engagement during lipophagy.	Validating models of LD budding from ER, verifying physical continuity *versus* close apposition at contact sites, and characterizing LD morphology in different metabolic states	Direct correlation of functional fluorescence readouts with high-resolution ultrastructure; powerful for mechanistic cell biology.	Fixed samples only; labor-intensive and low throughput; complex workflow and alignment procedures.
APEX-based proximity labeling at LDs	Engineered peroxidase (APEX/APEX2) is fused to an LD protein (*e.g.* PLIN2/3, DGAT2) and, in the presence of biotin-phenol and H_2_O_2_, biotinylates nearby proteins at the LD surface.	*In situ* LD-proximal proteome, including enzymes, trafficking factors, and transient interactors at LD surfaces and contact sites with mitochondria, ER, peroxisomes or lysosomes.	Mapping condition-specific LD interactomes (fed vs fasted, lipotoxic vs control, different tissues); identifying proteins that mediate LD-organelle contacts or LD remodeling.	Captures transient and weak interactions in living cells; preserves spatial context without the need to purify LDs.	Requires H_2_O_2_, which can induce oxidative stress; provides proximity rather than direct interaction information; careful controls (targeting-deficient or catalytically inactive APEX) are essential.
BioID/TurboID-based proximity labeling at LDs	Biotin ligases (BioID/BirA*, TurboID, miniTurbo) are fused to LD proteins and biotinylate neighboring proteins in living cells upon biotin supplementation.	LD-associated proteomes under defined metabolic conditions; identification of proteins stably residing at LDs or recurrently visiting LD surfaces.	Profiling LD proteomes in different cell types and tissues; comparing LD-proximal proteins between physiological and disease states (*e.g.* steatosis, cancer).	Operates under mild conditions without added oxidants; TurboID allows rapid labeling and dynamic studies of LD proteome remodeling.	Temporal resolution is limited by enzyme kinetics and biotin availability; biotin supplementation and overexpression may perturb cellular metabolism; proximity does not guarantee direct binding.
Split-TurboID or other split-proximity systems targeted to LDs and partner organelles	Complementary fragments of TurboID (or related enzymes) are targeted separately to LDs and another organelle (*e.g.*, mitochondria, ER, peroxisomes); active enzyme reconstitutes only when the two membranes are in sustained contact.	Proteomes specifically enriched at LD-mitochondria, LD-ER, or LD-lysosome contact sites, rather than at each organelle in isolation.	Dissecting the molecular composition of LD-organelle contact sites and how it changes with nutrient status, hormonal signals or pharmacological perturbation.	High specificity for true contact zones; enables direct comparison between different contact interfaces in the same cell type.	Requires co-expression and correct targeting of both fragments; contact-promoting constructs can potentially alter native contact dynamics; currently more technically specialized than single-enzyme proximity labeling.
Biochemical LD isolation + MS proteomics	LDs are purified by flotation on density gradients and analyzed by MS to determine their protein composition.	Global LD-associated proteome across tissues or conditions, including metabolic enzymes, structural proteins, trafficking machinery and signaling factors.	Building reference LD proteomes; comparing LD protein composition in adipocytes *versus* hepatocytes *versus* immune cells, or in healthy *versus* diseased states.	Unbiased and comprehensive view of LD-associated proteins; compatible with quantitative MS (label-free, SILAC, TMT) and integrative omics.	LD preparations can be contaminated with ER, mitochondria, or lysosomes; loss of spatial and temporal information compared with proximity labeling; requires relatively large material amounts.
Lipidomics of isolated LDs + stable isotope tracing	LD fractions are isolated and subjected to MS-based lipidomics; stable isotope–labeled precursors (*e.g.* fatty acids, acetate) are used to trace flux through LD lipid pools.	Detailed composition of TAG, sterol esters and associated phospholipids in LDs, and how these pools are remodeled in response to metabolic cues or disease.	Determining how LDs buffer specific lipid species, contributed to membrane synthesis, or supply substrates for β-oxidation; quantifying altered LD lipid flux in MASLD, cancer or lipodystrophy.	High chemical resolution and quantitative information; directly links LD biology to whole-cell lipid metabolism.	No single-droplet or spatial resolution; requires specialized MS infrastructure and careful experimental design; isotopic labeling schemes can be complex.
High-content imaging + automated LD quantification	Large-scale fluorescence imaging (LD dyes or FP markers) combined with automated segmentation and feature extraction (*e.g.* CellProfiler) to quantify LD phenotypes.	Population-level metrics of LD number, size, intensity and spatial distribution; indirect information on LD-organelle relationships *via* co-localization readouts.	Conducting genetic or small-molecule screens for regulators of LD biogenesis, expansion, lipolysis, lipophagy, or LD-organelle interactions.	As a scalable and objective, it is well suited for phenotypic screens and parameter sweeps; integrates readily with downstream omics on selected hits.	Limited mechanistic insight without complementary biochemical/proteomic data; co-localization metrics are correlative; analysis quality depends on segmentation and image quality.

### Fluorescent dyes and lipid probes

**Fig. 4:**
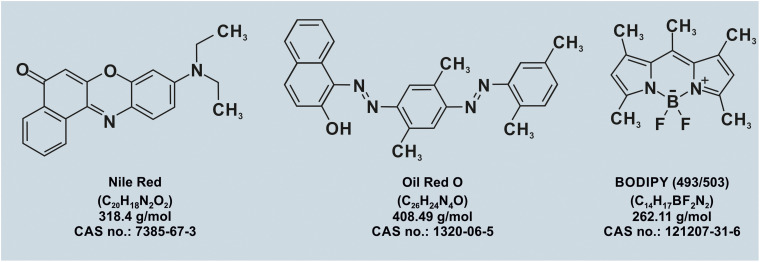
Chemical structures and key identifiers of neutral lipid-selective fluorescent dyes commonly used for lipid droplet imaging. The panel shows the structural formulas of Nile Red, Oil Red O, and BODIPY (493/503), along with their molecular formulas, molecular weights, and Chemical Abstract Service (CAS) numbers. These small-molecule probes selectively accumulate in the triacylglycerol and sterol ester-rich neutral lipid cores of lipid droplets. They are widely used in both fixed and live cells to quantify lipid droplet number, size, and subcellular distribution through confocal and high-content imaging.

Visualization of LDs in live and fixed cells has been greatly facilitated by neutral lipid-selective fluorescent dyes such as the classical Nile Red, Oil Red O (ORO) and BODIPY (493/503)-based probes (Fig. 4).^88^ These dyes partition into the hydrophobic LD core and exhibit strong fluorescence in a nonpolar environment. More recent probes have improved spectral properties, photostability, and selectivity, enabling multi-color imaging and super-resolution microscopy of LDs.^89–91^ Chemical biology approaches have yielded fluorescently tagged fatty acids, cholesterol analogs, and TAG mimetics that can be incorporated into LDs and tracked over time. For example, BODIPY-labeled fatty acids and click-chemistry-compatible alkyne fatty acids allow metabolic pulse-chase experiments that monitor LD formation, growth, and turnover. Photoactivatable and -switchable lipid probes further enable spatiotemporal control of lipid release and tracking of LDs.^92^ These tools permit quantitative analysis of LD dynamics, including rates of LD nucleation, fusion, fission, and lipolysis, as well as LD motility and interactions with other organelles in living cells.

### Proteomics and proximity labeling of lipid droplet proteins

Characterization of the LD proteome has been achieved through biochemical isolation of LDs followed by mass spectrometry (MS)-based proteomics. Early studies were hindered by contamination from other organelles, but improvements in LD isolation and MS technology have led to high-confidence catalogues of LD-associated proteins across various organisms and tissues.^93^

Proximity labeling techniques, such as those using engineered ascorbate peroxidase (APEX), proximity-dependent biotin identification (BioID), TurboID, or methods using genetically modified LD surface markers, super-resolution imaging techniques, or MS-based techniques have revolutionized the mapping of organelle-associated proteomes *in situ* (*cf.*Table 2).^94–99^ By directing these enzymes to the LD surface, researchers can biotinylate proteins in close proximity to LDs under specific conditions, enabling the dynamic profiling of the LD interactome during metabolic changes. These methods have unveiled transient LD interactions with enzymes related to lipid metabolism, vesicular trafficking, and signaling, as well as surprising LD associations of proteins traditionally linked to other compartments.

### Lipidomics and metabolic flux analysis

Lipidomics, the comprehensive analysis of lipid species by MS, provides detailed information on the composition of LDs and how it changes in response to metabolic stimuli or disease.^4,100^ Combining LD isolation with targeted or untargeted lipidomics enables quantification of TAG, sterol esters, and other neutral lipid species, as well as associated phospholipids. Stable isotope tracing with labeled fatty acids or precursors can be integrated with lipidomics to measure fluxes through lipid pathways and to distinguish between *de novo* synthesized lipids and those taken up from the environment.^101^ Such approaches have illuminated how LDs buffer specific lipid species, how they contribute to membrane lipid pools, and how lipid flux through LDs is rewired in metabolic diseases and cancer.

### Imaging lipid droplet-organelle contacts and dynamics

Advanced microscopy techniques have made it possible to visualize LD interactions with other organelles at high spatial and temporal resolution. Confocal and spinning-disk microscopy, combined with organelle-specific fluorescent markers, reveal dynamic LD-mitochondria, LD-peroxisome, and LD-ER contacts. Super-resolution techniques, such as Stimulated Emission Depletion (STED), Photoactivated Localization Microscopy (PALM), and Stochastic Optical Reconstruction Microscopy (STORM), have been applied to resolve the nanoscale organization of LD surfaces and contact sites.^102–104^ Moreover, correlative light and electron microscopy (CLEM) can link dynamic live-cell imaging with ultrastructural detail, helping to clarify the architecture of LD budding sites and contact zones.^105^ Quantitative image analysis, automated segmentation methods, and automated LD quantification systems support high-content screening of LD phenotypes and dynamics in response to genetic or chemical perturbations.^106^

### Genetic and small-molecule perturbations

CRISPR-based screening has identified numerous genes that influence LD formation, size, and turnover, including novel regulators of neutral lipid synthesis, LD budding, and contact site formation.^107–109^ These genetic perturbations provide mechanistic insight into LD biology and highlight potential therapeutic targets. Small molecules that modulate LD metabolism include inhibitors and activators of DGATs, ACAT/SOAT, ATGL, HSL, and other lipid-handling enzymes, as well as compounds that influence autophagy and lysosomal function.^110^ Chemical probes can acutely manipulate LD dynamics and thereby dissect causal relationships between LD remodeling and cellular signaling, viability, and metabolic outputs.^111^ Together, these chemical biology and systems-level approaches continue to deepen our understanding of LD dynamics and their roles in metabolic regulation.

### Therapeutic targeting of lipid droplet dynamics

LD biology offers multiple potential points of therapeutic intervention, which are summarized in Table 3. Strategies can aim to reduce pathological LD accumulation, enhance protective LD functions, or modulate LD-organelle coupling to restore metabolic balance.^112,113^ In conditions characterized by excessive lipid storage, such as MASLD, some forms of obesity, and neoplastic diseases, inhibitors of *de novo* lipogenesis and neutral lipid synthesis (*e.g.*, targeting acetyl-CoA carboxylase (ACC), fatty acid synthase (FASN), DGATs, or ACAT/SOAT) may reduce LD formation and limit hepatic or adipose steatosis.^110,113^ However, suppressing LD biogenesis without providing alternative routes for lipid handling risks the aberrant accumulation of FFAs exacerbating lipotoxicity.^28^ Consequently, successful approaches will need to finely tune LD formation relative to oxidation and export. Conversely, in lipodystrophy and other states of impaired safe storage, enhancing LD biogenesis and expansion in adipocytes may improve systemic metabolic parameters by increasing adipose storage capacity and reducing ectopic lipid deposition. Modulators of seipin, perilipins, and adipogenic transcription factors could contribute to such strategies, though specificity and safety remain major concerns.^16,114^ Targeting lipolysis and lipophagy provides additional levers. In obesity and type 2 diabetes, reducing excessive adipocyte lipolysis may help lower circulating FFA levels and insulin resistance.^115^ In heart failure or ischemia-reperfusion injury, facilitating LD mobilization could support energy production, presenting a potential target for cardiac dysfunction in conditions of obesity.^116^ Modulation of lipophagy may be beneficial in MASLD by enhancing hepatic LD clearance, but chronic activation of autophagy must be carefully controlled to avoid deleterious effects.^117^

**Table 3:** Representative therapeutic strategies targeting lipid droplet dynamics in metabolic disease

Strategy/class of intervention	Principal target(s)/pathway	Primary effect on LD dynamics	Main disease context(s)	Key opportunities/considerations
Inhibition of *de novo* lipogenesis and neutral lipid synthesis	ACC, FASN, DGAT1/2, ACAT/SOAT, and other related lipogenic enzymes	Decreases TAG and sterol ester synthesis, limiting LD formation and expansion in hepatocytes and adipocytes	MASLD/MASH, obesity, and certain cancers	Reducing pathological steatosis and LD-dependent tumor growth may be possible, but excessive inhibition could lead to the accumulation of toxic FFAs and membrane stress if alternative disposal routes are lacking.
Enhancement of adipose LD storage capacity for “safe storage”	PPARγ, other adipogenic factors, seipin, perilipins, and LD-stabilizing proteins	Increases LD biogenesis and stabilization in adipocytes, expanding safe neutral lipid storage	Lipodystrophies, partial lipodystrophy in metabolic syndrome, and ectopic steatosis	Lowering ectopic lipid deposition and improving systemic insulin sensitivity are benefits, but there is a risk of promoting weight gain or adipose expansion. Therefore, tissue-specific targeting is preferred.
Modulation of cytosolic lipolysis	ATGL, HSL, MGL, CGI-58, G0S2, and their associated regulators	Tunes TAG hydrolysis and FFA release from LDs in adipose and non-adipose tissues	Obesity, type 2 diabetes, cachexia, heart failure, and ischemia-reperfusion injury	Reducing excessive adipocyte lipolysis may improve insulin sensitivity. Enhancing LD mobilization can support cardiac or muscular energy demands, but both directions carry the risk of ectopic lipid overload or energy deficiency.
Modulation of lipophagy and autophagy	TFEB, mTORC1, core autophagy machinery including ATG proteins, and lysosomal acid lipase	Alters autophagic degradation of LDs, especially in hepatocytes and macrophages	MASLD/MASH, atherosclerosis, and cardiometabolic disease	Enhancing lipophagy can promote hepatic LD clearance and mobilize plaque lipids. However, chronic or uncontrolled autophagy can trigger cell death and inflammation. Therefore, careful temporal control is necessary.
Targeting LD-organelle contact sites	Tethers and scaffolds at LD-mitochondria and LD-peroxisome contacts such as PLIN isoforms, MIGA2, MFN2, Rab32, ACSL1, SNAP23, and PLIN1-MFN2 complexes)	Adjusts coupling of LD lipolysis to mitochondrial/peroxisomal β-oxidation, thermogenesis, and lipid detoxification	Skeletal muscle insulin resistance, obesity, brown/beige adipose thermogenesis, and certain cancers	Strengthening LD-mitochondria coupling in muscle or brown fat may enhance FA oxidation and energy expenditure. Weakening it in tumors could limit lipid fuel supply, but tissue-specific targeting is crucial to avoid systemic side effects.
Exploitation of LDs and LD-mimetic particles as delivery platforms	Engineered LD surface lipids and proteins, LD-targeting peptides and ligands, and LD-like neutral-lipid nanoparticles	Uses LD-like cores and surface modifications to encapsulate hydrophobic drugs and associate nucleic-acid cargos (*e.g.* miRNAs, siRNAs, mRNAs) for targeted delivery	Metabolic liver disease, cancer, inflammatory and fibrotic disorders	Conceptually related to lipid nanoparticle systems but leveraging endogenous LD pathways. There is potential for cell-type-specific targeting *via* LD-associated ligands, yet issues of biodistribution, immunogenicity, and interference with physiological LD functions must be carefully addressed.

Emerging approaches aim to selectively modulate LD-mitochondria or LD-peroxisome contact sites, altering lipid flux between storage and oxidation.^43,118^ For example, enhancing LD-mitochondria coupling in skeletal muscle could enhance FA oxidation and insulin sensitivity,^119^ while reducing such coupling in certain cancers might deprive tumor cells of lipid fuel or make cancer cells more susceptible to chemotherapy.^113^

Beyond manipulating LD dynamics *in situ*, LDs themselves and LD-mimetic particles may be exploitable as drug delivery platforms. Their neutral lipid core provides high loading capacity for hydrophobic small-molecule therapeutics, while the surrounding phospholipid monolayer and LD-associated proteins offer opportunities to display targeting ligands or antibodies that direct cargos to specific cell types or tissues.^119–121^ In principle, LD-like particles could also be engineered to carry nucleic acid therapeutics, including miRNAs and other small RNAs, by incorporating cationic or ionizable lipids and RNA-binding moieties at the LD surface. Such approaches conceptually extend clinically used lipid nanoparticle technologies by harnessing endogenous LD trafficking routes and contact sites, but they remain largely at the proof-of-concept stage. Systematic studies will be required to understand how LD-based carriers interact with innate immune pathways, how they distribute across organs, and how they can be designed so as not to disrupt the protective and homeostatic functions of physiological LDs.

A major challenge for LD-targeted therapies is achieving tissue specificity. Additionally, LD-mitochondrial interactions appear to be mediated by tissue-specific tethering complexes. These complexes include the small GTPase Rab32, the acyl-CoA synthetase ACSL1, together with the SNARE protein SNAP23. Other complexes involve p53-PLIN2 and PLIN5 in the liver, the outer mitochondrial membrane protein MIGA2, and a PLIN1-mitofusin-2 (MFN2) complex in brown adipocytes. In skeletal muscle, interactions involve PLIN5-Rab8A and PLIN2, among others.^122^ Because LDs are present in all tissues and have both protective and potentially harmful functions, systemic manipulation of LD dynamics poses the risk of unintended consequences in unaffected tissues. Targeted delivery, cell type-specific gene modulation, and context-dependent pharmacology will be crucial for translating LD biology findings into safe and effective therapies.

## Conclusions and outlook

LDs have emerged as central integrators of cellular and systemic metabolism. Their dynamics, including biogenesis, growth, remodeling, interactions with other organelles, and catabolism, are finely tuned to balance energy storage with energy expenditure, protect cells from lipotoxicity, and support adaptation to environmental and physiological challenges. Tissue-specific LD programs in adipose tissue, liver, skeletal muscle, pancreatic β-cells, and other organs collectively shape whole-body energy homeostasis and the response to nutritional and hormonal signals. Disruption of these programs underlies a wide array of metabolic diseases, including obesity, insulin resistance, MASLD, lipodystrophies, and atherosclerosis, as well as contributing to cancer and other pathologies. Recent advances in chemical biology, imaging, lipidomics, and proteomics have provided powerful tools to study LD dynamics at unprecedented spatial and temporal resolution, revealing complex LD-organelle networks and regulatory circuits. These technologies are beginning to uncover molecular principles that can be exploited for therapeutic intervention, whether by modulating LD formation, lipolysis, lipophagy, or organelle contacts. Despite this progress, many questions remain. How are LD budding sites selected and organized at the ER? What dictates the specificity and plasticity of LD contact sites with different organelles? How do cells integrate signals from metabolic and stress pathways to coordinate LD dynamics with global cellular and organismal needs? And how can we safely manipulate LD biology in a tissue- and context-dependent manner for therapeutic benefit? An additional, relatively unexplored avenue is to harness LD-like particles as delivery vehicles for small-molecule drugs and nucleic acid therapeutics. This builds on their intrinsic capacity to package lipids and interface with multiple organelles.

Answering these questions will require the continued integration of mechanistic biochemistry, cell biology, physiology, and systems-level approaches. As our understanding deepens, LDs will increasingly be regarded not merely as passive fat stores but as dynamic organelles whose regulated behavior is fundamental to metabolic health. They may represent ideal therapeutic targets across various human disorders.

## Author contributions

R. W. conceived the topic and overall structure of the review. R. W. and A. L. performed the literature survey and wrote the first draft of the manuscript. R. W., S. W. and A. L. contributed to the organization of the sections, revised the text critically for important intellectual content, and approved the final version of the manuscript. All authors agreed on the final content and are accountable for all aspects of the work.

## Conflicts of interest

There are no conflicts to declare.

## Data Availability

No new data were generated or analysed in this review. All data discussed are drawn from previously published studies cited in the Notes and references section.
